# Tumor Flare Reaction in a Classic Hodgkin Lymphoma Patient Treated With Brentuximab Vedotin and Tislelizumab: A Case Report

**DOI:** 10.3389/fimmu.2021.756583

**Published:** 2022-01-14

**Authors:** Chunting Zhu, Yi Zhao, Fang Yu, Weijia Huang, Wenjun Wu, Jingsong He, Zhen Cai, Donghua He

**Affiliations:** ^1^ Bone Marrow Transplantation Center, The First Affiliated Hospital, College of Medicine, Zhejiang University, Hangzhou, China; ^2^ Pathology Department, The First Affiliated Hospital, College of Medicine, Zhejiang University, Hangzhou, China

**Keywords:** brentuximab vedotin, tislelizumab, tumor flare reaction, classic Hodgkin lymphoma, immune related adverse event

## Abstract

**Background:**

Tumor flare reaction (TFR) is a clinical syndrome, which is mainly associated with painful and swollen lymph nodes or splenomegaly, slight fever, bone pain, and skin rash during treatment with immune-related drugs, causing difficulty in distinguishing TFR from disease progression. Brentuximab vedotin (BV) and programmed death 1 (PD-1) inhibitor are two ideal drugs used for the treatment of classic Hodgkin lymphoma, but few studies have reported their adverse effects in association with TFR. The efficacy and safety of monotherapy or combination therapy with these drugs needs to be further evaluated. It is essential to determine whether treated patients can develop TFR, thus enabling more accurate diagnosis and treatment.

**Case presentation:**

A 26-year-old female patient, diagnosed with classic Hodgkin lymphoma, had received 2 + 3 cycles of ABVD chemotherapy (a combination of adriamycin, bleomycin, vinblastine, and dacarbazine) and 4 cycles of PD-1 inhibitor (tislelizumab) therapy but exhibited poor efficacy. Subsequently, she was given combination therapy of BV (100 mg) + tislelizumab (200 mg). However, a slight fever, painful and swollen axillary lymph nodes, multiple skin rashes with pruritus, joint pain, and fatigue with poor appetite appeared during the treatment. Ultrasound (US) scans revealed that multiple lymph nodes were significantly enlarged. After treatment with low-dose dexamethasone and cetirizine, the symptoms were alleviated. A biopsy of the left axillary lymph node revealed that lymphoid tissue exhibited proliferative changes, without tumor cell infiltration. These findings were consistent with the clinical and pathological manifestations of TFR.

**Conclusion:**

Combination therapy with BV and PD-1 inhibitor was effective in the treatment of relapsed or refractory classic Hodgkin lymphoma. The results suggest that the combination therapy may cause TFR, and biopsy and also continuous imaging observation are important to determine the disease stage. This approach allows clinicians to decide whether to continue the current treatment plan, and alerts them to the occurrence of excessive activation of the immune system.

## Introduction

Brentuximab vedotin (BV) is a biological agent with an immune function, which is composed of three components, namely, an anti-CD30 monoclonal antibody(cAC10), a potent antimicrotubule agent (monomethyl auristatin E, MMAE), and a dipeptide linker that can be cleaved by proteases in lysosomes (1, 2). In the human body, after binding to CD30 on the cell surface, BV is internalized and the linker is cleaved to release MMAE, exerting its cytotoxic effect. Then, MMAE can inhibit tubulin aggregation, disrupt the intracellular tubulin skeleton and arrest the cell cycle (G2/M phase) inducing the apoptosis of the target cells (3). The induction of apoptosis by the anti-tubulin action of MMAE, and activation of the innate immune system by an antitumor immune reaction that induces immunogenic cell death through endoplasmic reticulum stress are two major molecular mechanisms of BV (4). BV as monotherapy or in combination with chemotherapy has been reported to produce satisfactory outcomes in patients with relapsed or refractory Hodgkin lymphoma (5–8). Common BV-associated adverse effects include neutropenia, thrombocytopenia, peripheral sensory neuropathy, fatigue, rash, fever, constipation, nausea, poor appetite, and infection (1, 3, 4, 9–12).

Programmed death 1 (PD-1) inhibitor, a human or humanized IgG4 monoclonal antibody, is an immune checkpoint inhibitor that has shown promise for the treatment of relapsed or refractory Hodgkin lymphoma (8, 13). However, fatigue, poor appetite, rash, pruritus, diarrhea, nausea and infection are common adverse effects (14). Immune associated adverse effects have also been reported, namely, hypothyroidism, pneumonia, hepatitis, colitis and skin rash (15, 16), with tumor flare reaction (TFR) also recognized as one of complications (17, 18). Tislelizumab is a humanized IgG4 anti-PD-1 monoclonal antibody and its adverse effects are similar to those elicited by nivolumab or pembrolizumab (16).

Targeted therapy has been widely used for the treatment of numerous malignant tumors. CD30 and PD-1 are two ideal therapeutic targets for classic Hodgkin lymphoma, and a number of relevant clinical trials have been carried out (1, 2, 7, 8). The efficacy of combination therapy with the two drugs is still under evaluation, with reported common adverse effects at present being fatigue, nausea, rash, pruritus, vomiting, diarrhea, and infusion-related adverse reactions (4). That a PD-1 inhibitor can cause TFR has been previously reported (18), but there are few reports on TFR associated with BV (1, 19). One was reported in a phase II study of relapsed/refractory systemic anaplastic large-cell lymphoma with BV that 4 patients experienced painful enlargement of lymph nodes and erythema after the administration of BV, without pathological biopsy of the enlarged lymph nodes (1). Another study described 2 patients who experienced TFR to BV after Lenalidomide treatment (19). However, there are no specific reports of TFR caused by BV with PD-1 inhibitor treatment of hematological malignancies. The present study reports a case of TFR in a patient who received combined administration of BV and tislelizumab and explores the possible mechanism and diagnostic significance of TFR caused by immune-related adverse effects of drugs.

## Case Presentation

**Figure 1:**
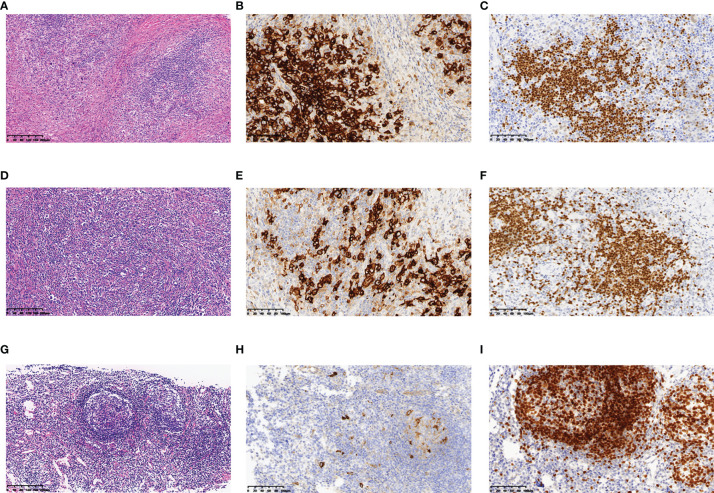
Histopathology and immunohistochemistry of the lymph nodes of this patient. In October 2019, **(A)** HE staining revealed nodular sclerosis classic Hodgkin lymphoma (10×), IHC staining showed that lymphoma cells were positive for **(B)** CD30 (20×), **(C)** PAX-5 (20×). In May 2020, **(D)** HE staining revealed nodular sclerosis classic Hodgkin lymphoma (10×), IHC staining showed that lymphoma cells were positive for **(E)** CD30 (20×), **(F)** PAX-5 (20×). In December 2020, **(G)** HE staining revealed lymphoid tissue had a proliferative change (10×), IHC staining showed that lymphoma cells were negative for **(H)** CD30 (20×), and positive for **(I)** PAX-5 (20×).

**Figure 2:**
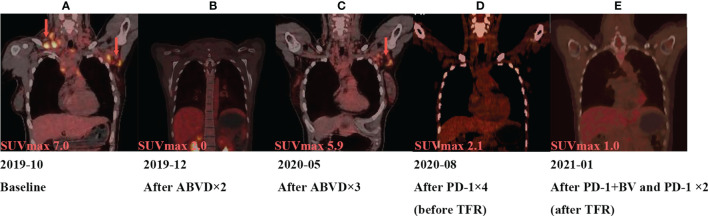
Tumor assessment during treatment by PET. **(A)** In October 2019, the size of the left axillary lymph node was 3.1 × 2.2 cm, and maximum standard uptake value (SUVmax) from 18F-FDG is 7.0. **(B)** In December 2019, the size of the left axillary lymph node was 1.51 × 0.84 cm, SUVmax is 2.0. **(C)** In May 2020, the left axillary lymph node was1.69 × 1.69 cm, and SUVmax is 5.9. **(D)** In August 2020, the left axillary lymph node was 1.5 cm, and SUVmax is 2.1. **(E)** In January 2021, PET/CT showed the SUVmax is 2.1.

**Figure 3:**
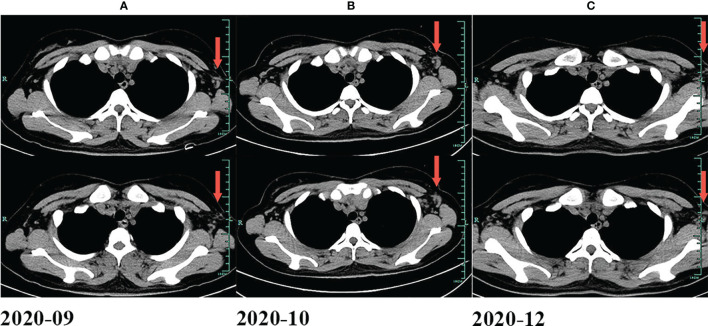
Changes of lymph nodes evaluated by CT. **(A)** In September 2020. **(B)** In October 2020. **(C)** In December 2020.

**Figure 4:**
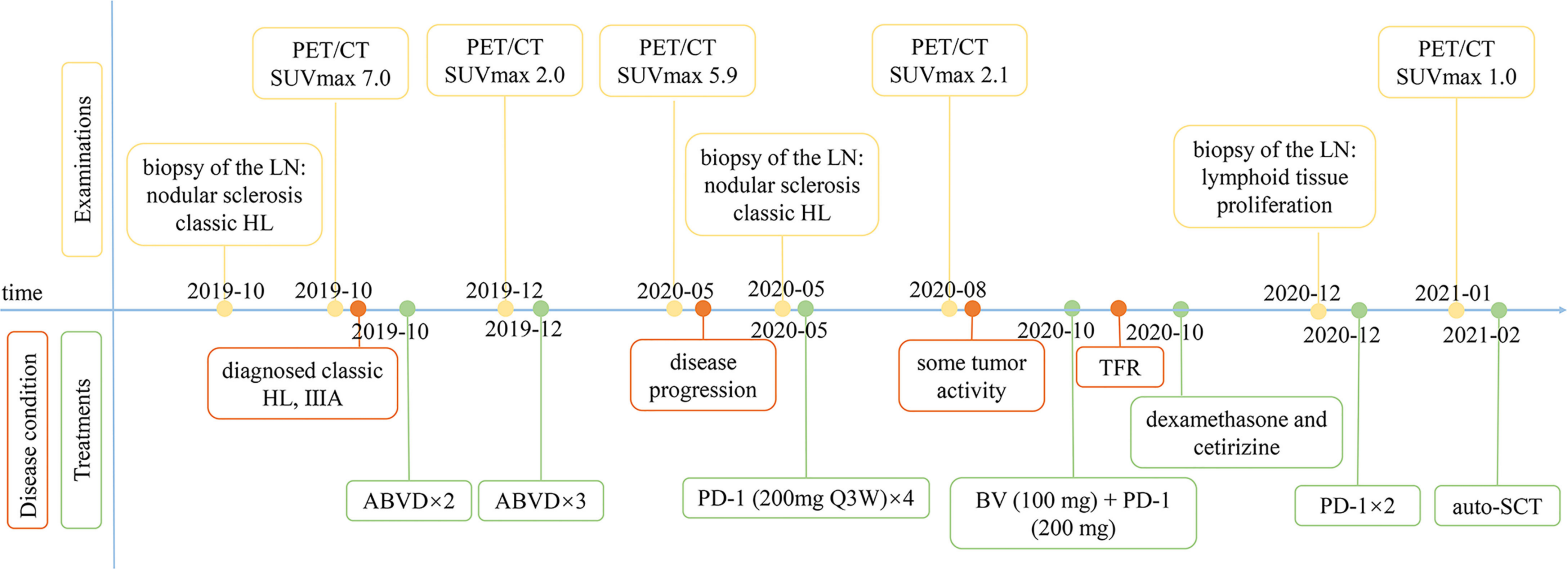
Clinical course of the patient. US, ultrasound; LN, lymph node.

A 26-year-old female patient was admitted to the First Affiliated Hospital of Zhejiang University School of Medicine (Hangzhou, China) in October 2019 due to persistent pain of the right side of the neck and left side of the axilla lymphadenopathy for more than 10 days. The lymph node on the right side of the neck was biopsied, and the pathology suggested nodular sclerosis classic Hodgkin lymphoma (**Figure 1A**), a subtype of classic Hodgkin lymphoma. Immunohistochemistry (IHC) results of CD30 (+) **(** **Figure 1B**), CD15 (+), PAX5 (+) (**Figure 1C**), Bcl-2 (+), MUM-1 (+), Bcl-6 (partial +), CD21 (FDC+), Ki-67 (+, 60%), PD-1 (small lymphocytes +, 20%), CD3, CD5, CD7, CD20, CD45, anaplastic lymphoma kinase (ALK), and EMA were all negative. There was no obvious abnormal lymphocyte group in bone marrow smears or after bone marrow immunophenotyping. A bone marrow biopsy revealed that the proliferation of hematopoietic tissue was active. The chromosomal analysis showed 46, XX (20). In October 2019, primary positron emission tomography-computed tomography (PET/CT) showed that there were multiple enlarged lymph nodes in the bilateral neck, bilateral inguen, and left axilla, and that the size of the left axillary lymph node was 3.1 × 2.2 cm (**Figure 2**). The patient was diagnosed as having nodular sclerosis classic Hodgkin lymphoma IIIA, and she subsequently received ABVD chemotherapy (a combination of doxorubicin, bleomycin, vinblastine, and dacarbazine) for 2 cycles. In December 2019, the secondary PET/CT showed that the size of the left axillary lymph node was significantly smaller and that glucose metabolism of FDG was reduced (**Figure 2**). The patient subsequently received 3 cycles of ABVD chemotherapy. In May 2020, a tertiary PET/CT examination showed that the left axillary lymph node was larger and that metabolism was increased (**Figure 2**), following local recurrence after treatment. A biopsy of the left axillary lymph node was undertaken, and the pathology indicated classic Hodgkin lymphoma, nodular sclerosis type (**Figure 1D**). The IHC results of testing of CD30 (+) (**Figure 1E**), CD15 (+), PAX-5 (+ weak) (**Figure 1F**), Bcl-2 (+), MUM1 (+), CD21 (FDc+), Ki-67 (+80%), Bcl-6, CD3, CD5, CD7, CD20, CD45, ALK, EMA, and EBER were all negative. Considering the disease progression of the patient, she received tislelizumab (200 mg Q3W) for 4 cycles. During that period, the left axillary lymph node of the patient was reduced in size. Next, the patient was scheduled to receive auto-SCT. She underwent the PET/CT in August, and we mobilized and collected autologous stem cells in September, without continuing tislelizumab treatment. In August 2020, the quaternary PET/CT revealed that multiple lymph nodes in the bilateral neck, the left clavicle and left axilla area were slightly enlarged and exhibited mildly increased metabolic activity (**Figure 2**) suggesting that there was still residual tumor activity after targeted therapy. Thus, combined therapy of BV (100 mg) + tislelizumab (200 mg) was administered to the patient in October, 2020. Before the treatment, the lymph nodes of the patient were not significantly changed as revealed by US and CT scans. Overall, the patient did not exhibit disease progression from August to the time she received combination therapy in October. However, the patient had a slight fever, with the highest temperature being 37.6 °C after 2 days of this round of treatment, experienced pain in the axillary lymph nodes and had multiple skin rashes with pruritus all over the body after 1 week, accompanied by joint pain, fatigue and poor appetite for about 15 days. Before these reactions, the patient had no history of autoimmune or hyperimmune reactivity. Testing for the presence of inflammatory cytokines revealed that interleukin-4 (IL-4) concentrations decreased from 3.41 to 0.1 pg/ml, IL-2 and tumor necrosis factor-α (TNF-α) from 1.54 or 4.13 to 0.1 pg/ml, respectively. Interferon-γ (IFN-γ) concentrations increased from 2.95 to 15.43 pg/ml, IL-6 from 8.15 to 12.96 pg/ml, and IL-17A from 51.12 to 176.68 pg/ml (**Table 1**). The US and CT scans showed that multiple lymph nodes were notably enlarged all over the body compared to before treatment (**Figure 3**). Next, the patient was given dexamethasone (5 mg i.v. QD) for 4 days combined with cetirizine (10 mg p.o. QD) for 3 days. The body rash of the patient subsided and pruritus was alleviated. During December, 2020, US scans revealed that the sizes of the bilateral cervical, axillary and inguen lymph nodes were reduced. A biopsy of the left axillary lymph node showed that lymphoid tissues exhibited a proliferative change (**Figure 1G**). The results of IHC indicated that CD30 (−) (**Figure 1H**), CD3 (+), CD20 (+), Ki-67 (around 5%+), CD5 (+), CD10 (germinal center +), Bcl-2 (+), Bcl-6 (germinal center +), MUM1 (scattered W+), PAX-5 (+) (**Figure 1I**), CD21 (FDC+), VD23 (FDC+), cyclinD1c-Myc, ALK and EBER were all negative. During the wait for auto-SCT, the patient received a further 2 cycles of PD-1. In January 2021, the quinary PET/CT scans showed there was no obvious increase in glucose metabolism of FDG in the area of the lymph nodes, after the lesion had shrunk and tumor activity inhibited(**Figure 2**). Then the patient underwent auto-SCT. At present, she has been regularly followed-up and remains disease free for 10 months since transplant without the need for anti-tumor treatment. The patient never received radiation therapy. The clinical course of the patient is shown in **Figure 4**.

**Table 1:** Changes of inflammatory cytokines.

Date	IL-2 (pg/ml)	IL-4 (pg/ml)	IL-6 (pg/ml)	IL-10 (pg/ml)	IL-17A (pg/ml)	TNF-α (pg/ml)	IFN-γ (pg/ml)
2020-09-01 (Before TFR)	1.54	3.41	8.15	2.88	51.12	4.13	2.95
2020-10-30 (TFR)	0.1	0.1	12.96	2.74	176.68	0.1	15.43
2020-12-04 (After TFR)	1.77	3.8	4.23	2.82	59.61	4.18	6.69

IL, interleukin; TNF-α, tumor necrosis factor-α; IFN-γ, interferon-γ.

## Discussion

TFR, the pseudoprogression of hematological malignancies, is a clinical syndrome caused by drugs with actions on the immune system, and often occurs in patients who are diagnosed with chronic lymphocytic leukemia (20), mantle cell lymphoma (21), Hodgkin lymphoma (22) or Waldenström’s macroglobulinemia (23, 24). TFR often manifests as painful and enlarged lymph nodes or splenomegaly in patients following treatment with immune-related drugs. It may be accompanied with symptoms of fever, bone pain, skin rash or lymphocytosis (25, 26), which are mainly mild and self-limiting and often appear during the initial phase of treatment. The course of the disease generally evolves over 7–14 days (27). In most cases, a patient with TFR can be given non-steroidal anti-inflammatory drugs to relieve the discomfort (25). But if the patient has severe symptoms or a history of immune checkpoint inhibitor related adverse effects, corticosteroid should be administered, and therapy stopped if necessary (21, 25). It may not be suitable to use multiple immune-related drugs at the same time, for example two immune checkpoint inhibitors.

In the present study, the patient neither had obvious discomfort after 4 treatments of intravenous tislelizumab infusion, nor significantly larger lymph nodes according to the results of PET/CT scans. After the first administration of BV combined with tislelizumab, low-grade fever, swollen and painful lymph nodes, multiple skin rashes with pruritic, joint pain, fatigue and poor appetite appeared. The symptoms of the patient improved after treatment with glucocorticoid and antihistamine therapy, which was consistent with the clinical manifestations of TFR. US and CT scans showed that the lymph nodes were significantly enlarged after combination therapy of BV and tislelizumab, and re-examination showed that multiple lymph nodes were significantly reduced with no anti-tumor treatment. These findings were in line with pseudoprogression determined retrospectively through imaging observations (28). The medical team considered the reaction to be TFR and decided to give the patient auto-SCT. As hospital beds were scarce in our center, the patient continued PD-1 treatment at home during the waiting time. Before retreatment of PD-1, US scans showed that there were still a number of swollen lymph nodes in the axilla and neck regions. In order to determine whether the patient was progressing or had TFR, we took a lymph node biopsy, which showed proliferative changes but no tumor cell infiltration was detected. In retrospect, it was indeed a pity we did not to take a lymph node biopsy during the onset of TFR.

The specific mechanism of the TFR remains to be elucidated, but may be related to the excessive activation of the immune system (25, 29) and the secretion of inflammatory cytokines (30). In the present study, the significant change of concentration of IL-17A might indicate the occurrence of inflammation, but other concentrations were still within the reference range. In addition, TFR is correlated with the activation and infiltration of NK cells and T cells in cancer foci and changes in the tumor microenvironment (26, 29). However, the biopsy of lymph node did not reveal infiltration of NK or cytotoxic T cells on the day 20 of treatment in a case that was TFR associated with lenalidomide in a follicular lymphoma patient (31). The pathophysiological changes of related lymph nodes or involved lesions after the occurrence of TFR have not been fully clarified, while the rapid appearance may indicate that the initial stage of TFR is mediated by non-antigen-specific effectors, and the later stage antigen-specific immune effects (29).

TFR is an important component of the antileukemic effect of lenalidomide. Chanan-Khan et al. pointed out that the intensity of TFR after the administration of lenalidomide in chronic lymphocytic leukemia patients was related to the complete response rate (25). To the best of our clinical knowledge, it is difficult to distinguish TFR or pseudoprogression from disease progression by one measurement of tumor size or metabolism using immediate imaging examinations. Skoura et al. reported a case of false-positive 18F-FDG PET/CT after rituximab therapy, the patient was finally diagnosed with TFR. PET/CT examination of that patient revealed increased metabolic activity of enlarged lymph nodes after R-CHOP (rituximab plus cyclophosphamide, doxorubicin, vincristine, and prednisone) treatment and allogeneic transplantation, whereas biopsy of the lymph node revealed extensive reactive T cell infiltration, with no signs of lymphoma cells. Re-examination of PET/CT scans showed no obvious enlargement or increased metabolic activity of lymph nodes after 3 months (32). Isolated measurements of the sizes of lymph nodes by US, CT or other imaging methods to evaluate changes in the severity of lymphoma are imprecise, and even the use of single PET/CT for the assessment for the changes of disease does not provide an accurate diagnosis, leading to difficulty in distinguishing TFR from disease progression and whether to terminate the original effective treatment therapy.

BV-related immune complications are not common, but include progressive multifocal leukoencephalopathy and acute pancreatitis (33, 34). A case of a Hodgkin lymphoma patient treated with BV, who developed progressive multifocal leukoencephalopathy, indicated that BV can reduce the number of CD30^+^ T cells, which may be related to immune surveillance, and also inhibition of the TNF signaling pathway (33). Additionally, Gandhi et al. demonstrated that low CD30 expression was detected in the pancreas of a patient and two healthy controls by multispectral imaging, suggesting that BV targeted to the unexpected low-level CD30^+^ pancreas may be the foundation of this adverse effect (34). A limited number of reports on TFR induced by BV exist, but the underlying mechanisms are still unclear. In a clinical study of BV combined with nivolumab for relapsed or refractory Hodgkin lymphoma patients, BV or nivolumab was administered on day 1 or day 8 in the treatment first cycle (4). For our patient, the two immune-related targeted drugs were simultaneously used during the first cycle, which may have triggered the TFR of the patient. In addition, CD30 is a member of the TNF receptor family and TNF-α can induce the nuclear factor-κB (NF-κB) pathway and regulate the local microenvironment to enhance intracellular killing (33). Therefore, BV-induced TFR may be related to the CD30 and TNF pathway, but further research is required to verify unequivocally this hypothesis.

In case of abnormal conditions, such as rapid enlargement of lymph nodes during the administration of immune-related therapeutic drugs or targeted therapies, clinicians should be additional aware of the occurrence of TFR that may be related to excessive activation of the immune system. In this clinical research, combination therapy using BV and tislelizumab may cause TFR. Thus, biopsy and continuous imaging observation are important to indicate the disease changes, enabling clinicians to determine the disease stage and real effects of the drugs of a patient, to facilitate the development of further accurate diagnostic and treatment schemes.

## Data Availability Statement

The original contributions presented in the study are included in the article/supplementary material. Further inquiries can be directed to the corresponding author.

## Ethics Statement

The studies involving human participants were reviewed and approved by the Clinical Research Ethics Committee–IIT Ethics Review Group, The First Affiliated Hospital, College of Medicine, Zhejiang University. The patients/participants provided their written informed consent to participate in this study.

## Author Contributions

DH and WH carried out the clinical management of the patient. FY and WW reviewed and analyzed the data. CZ and YZ wrote the manuscript. JH and ZC supervised the entire study. All authors contributed to the article and approved the submitted version.

## Funding

This work was supported by the National Natural Science Foundation of China (81770217), and the Natural Science Foundation of Zhejiang Province (LY16H080001).

## Conflict of Interest

The authors declare that the research was conducted in the absence of any commercial or financial relationships that could be construed as a potential conflict of interest.

## Publisher’s Note

All claims expressed in this article are solely those of the authors and do not necessarily represent those of their affiliated organizations, or those of the publisher, the editors and the reviewers. Any product that may be evaluated in this article, or claim that may be made by its manufacturer, is not guaranteed or endorsed by the publisher.
